# Telehealth Usage Disparities in Israel in Light of the COVID-19 Pandemic: Retrospective Cohort Study of Intersectional Sociodemographic Patterns and Health Equity Implications

**DOI:** 10.2196/77600

**Published:** 2025-11-27

**Authors:** Motti Haimi, Efrat Shadmi, Tzipi Hornik-Lurie, Daniel Sperling

**Affiliations:** 1 Health Systems Management Department, Max Stern Academic College of Emek Yezreel, D. N Emek Yezreel, Emek Yezreel, 1930600, Israel, 972 46423504, 972 72334523; 2 Faculty of Medicine, Technion – Israel Institute of Technology, Haifa, Israel; 3 Clalit Health Services, Tel Aviv, Israel; 4 Department of Nursing, University of Haifa, Haifa, Israel; 5 Research Room, Meir Medical Center, Kfar Saba, Israel

**Keywords:** equity, digital health, telehealth, intersectionality, after-hours

## Abstract

**Background:**

Telehealth has become a transformative health care delivery approach post the COVID-19 pandemic. Although telehealth improves health care access and reduces disparities, mounting evidence suggests usage patterns may exacerbate pre-existing health care inequities. Understanding these patterns across diverse populations is crucial for equitable digital health implementation.

**Objective:**

This study aimed to examine telehealth usage patterns across sociodemographic groups in Israel’s universal health system to identify equity issues. We investigated variations across intersecting demographic characteristics during pre-, mid-, and post-COVID-19 periods and assessed evolving after-hours usage patterns.

**Methods:**

We conducted a retrospective cohort analysis using health and administrative data from the electronic database of Clalit Health Services’ Sharon-Shomron District in Israel. The study population comprised 499,607 adult members (≥25 years; mean age 50.6, SD 16.5 years) with continuous enrollment from March 2019 to February 2022. We analyzed telehealth usage across 3 periods that are pre-COVID-19 (March 2019-February 2020), COVID-19 (March 2020-February 2021), and post-COVID-19 (March 2021-February 2022). Telehealth services included telephone consultations, video consultations, and TYTO (Tytocare) remote diagnostic device usage. Primary outcomes were telehealth usage rates and after-hours usage patterns. We used descriptive statistics, temporal trend analysis, and multivariable logistic regression with bootstrapping.

**Results:**

Telehealth usage among unique members more than doubled from 4.06% (20,264/499,607) pre-COVID-19 to 9.38% (46,868/499,607) post-COVID-19. Significant intersectional disparities emerged across multiple dimensions. In the post-COVID-19 period, young adults (25‐35 years) used telehealth at 3.1 times the rate of older adults (≥70 years; 18,333/102,533, 17.9% vs 4129/72,280, 5.7%). Women consistently showed higher usage than men (26,702/258,471, 10.3% vs 20,166/241,136, 8.4% post-COVID-19). Profound socioeconomic disparities persisted, with high socioeconomic status members using telehealth at nearly 4 times the rate of low socioeconomic status members (19,064/172,011, 11.1% vs 1328/56,154, 2.4% post-COVID-19). Cultural differences were striking: religious Jewish sector members demonstrated nearly 10-fold higher usage than Arab and Bedouin members (904/7630, 11.8% vs 1125/76,895, 1.5% post-COVID-19). A U-shaped relationship with peripherality (geographic distance from major urban centers and service availability) persisted after adjusting for socioeconomic status. In geographic analyses, this pattern remained across locations. After-hours telehealth usage declined from 65% (324,744/499,607) of all telehealth visits pre-COVID-19 to 49% (244,807/499,607) post-COVID-19, indicating telehealth’s evolution from an after-hours alternative to an integrated health care component. Multivariable analysis confirmed these disparities remained significant after adjusting for demographic and health factors.

**Conclusions:**

Telehealth expansion benefits remain unevenly distributed across populations in Israel’s universal health care system. Significant disparities along age, socioeconomic, cultural, and geographic lines suggest that digital health innovations may widen existing health care inequities without interventions. Intersectional disparities require multidimensional approaches to overlapping barriers. Health care systems must intentionally address equity considerations to ensure digital health and telehealth integration reduces, not worsens, existing health care disparities in routine care delivery.

## Introduction

### Background

Telehealth has emerged as a transformative approach to health care delivery, particularly accelerated by the COVID-19 pandemic [1,2]. While this digital modality offers potential solutions to health care access challenges by connecting patients with providers regardless of geographic constraints, growing evidence suggests that telehealth adoption and usage patterns reflect and potentially amplify existing disparities within health care systems [3,4].

The concept of telehealth—the delivery of health care services through information and communication technologies across distance [5]—has transformed from a convenient alternative to a fundamental component of modern health care systems [6]. This digital transformation promises to revolutionize health care delivery by offering patients real-time clinician access without physical clinic visits [7,8], potentially increasing care access and reducing health disparities among rural and underserved populations [9-11].

Telehealth provides substantial benefits, particularly for nonemergency care, reducing health center resource usage while improving access and ensuring care continuity [12,13]. It offers expanded access to specialists and demonstrates cost savings with equal or superior care quality compared to traditional care models [14-18]. The COVID-19 pandemic triggered exponential implementation, making telehealth exceptionally valuable during mandated social isolation [19]. Although in-person visits have resumed, telemedicine remains integral to health care delivery [20]. However, significant barriers persist for technologically challenged individuals, those with limited health literacy, and patients experiencing operational difficulties [21-23].

The rapid telehealth expansion raises critical equity concerns. People with limited digital literacy, inadequate access to digital devices or reliable internet, and those with limited language proficiency face unique challenges. Research confirms that racial and ethnic minorities, lower-income individuals, and rural residents have significantly lower broadband access rates [24,25]—a prerequisite for effective telehealth engagement.

Health equity, defined as the absence of avoidable and unfair health differences between population groups [26], stands at the center of telehealth evaluation. Equity in telehealth requires acknowledging that different populations may need different levels of support to achieve comparable health care outcomes [27,28], contrasting with equality’s emphasis on uniform resource distribution [29,30]. The World Health Organization identifies complex social determinants of health that significantly impact health outcomes [31].

The concept of intersectionality [32] provides a critical framework for understanding how overlapping sociodemographic characteristics create unique experiences of advantage or disadvantage in telehealth access [33,34]. When applied to telehealth, intersectionality reveals that factors such as age, race or ethnicity, gender, socioeconomic status (SES), and geographic location interact in complex ways that influence health care engagement patterns [27-29,35,36].

### Global Perspectives and Intersectional Frameworks

Research has identified concerning patterns in telehealth adoption: lower usage among older adults [24,37], racial and ethnic minorities [38,39], individuals with lower incomes [40,41], those with limited English proficiency [42,43], and rural residents [44,45]. These disparities correlate with broader health care service usage differences and health outcomes [24,46].

The digital divide significantly influences telehealth usage patterns. Research by Perzynski et al [47] found that patients with limited digital access were less likely to use patient portals, while Ramsetty and Adams [48] documented how technological barriers intersect with social determinants of health to exacerbate health care disparities. These technological disparities cannot be viewed in isolation from cultural, linguistic, and socioeconomic factors [49-51].

Recent US research reveals troubling patterns: ethnic minorities use telehealth significantly less than White populations [52], with telehealth widening gaps in sexual and reproductive health services [53]. Even when used, telehealth effectiveness varies across demographic groups, with documented racial disparities in diabetes management outcomes [54] and socioeconomic digital divides in primary care settings [55].

Current literature frequently examines sociodemographic factors in isolation rather than considering intersectional effects [56,57], failing to capture how multiple dimensions combine to create unique barriers [58,59]. Recent work by Husain and Greenhalgh [60] and Velasquez and Mehrotra [61] has begun applying intersectional frameworks to reveal nuanced adoption patterns. The COVID-19 pandemic has highlighted these disparities [62,63], with evidence showing varied telehealth experiences based on intersecting identities [64-66].

### Israeli Context

Examining telehealth equity in Israel is particularly crucial. The Israeli health care system operates under the National Health Insurance Law, ensuring all residents receive standardized health care services through 4 nonprofit Health Funds. Clalit Health Services covers nearly 50% of the population [67]. Despite universal coverage, widening health care access gaps affect non-Jewish populations, economically disadvantaged groups, and geographically peripheral residents [68].

Recent Israeli research documents significant telehealth disparities. Reges et al [69] found ethnicity as the most discriminatory predictor of telemedicine use, with Jews and Arabs accounting for 85% and 52% of users, respectively. Penn and Laron [70] identified compounded barriers among Arab Israeli women older than the age of 65 years, including a lack of awareness, lower digital literacy, and language barriers [70]. Geographic disparities between central urban areas and peripheral communities, particularly affecting Bedouin populations, have been documented [71].

### Research Question and Purposes

This study examines health equity considerations in telehealth usage, focusing on intersections between ethnic background, geographic location, and SES. We seek to identify disparity patterns and inform policies ensuring telehealth expands rather than restricts health care access.

The study addresses how telehealth services are used among population groups characterized by combined social determinants of health. Our objectives are to (1) describe sociodemographic and health characteristics of telehealth users and service implications, (2) examine whether telehealth implementation reflects health care access gaps, (3) analyze after-hours telehealth usage patterns to understand telehealth’s evolving role in health care delivery and accessibility, and (4) identify strategies to improve telehealth accessibility across diverse populations.

## Methods

### Study Design and Setting

This retrospective cohort study analyzed telehealth usage patterns among adult members of Clalit Health Services (CHS) in Israel’s Sharon-Shomron District. CHS is Israel’s largest health fund and integrated delivery system, serving nearly 50% of the Israeli population under the National Health Insurance Law. The organization functions as both insurer and provider, directly operating hospitals and clinics while ensuring universal health care coverage.

The Sharon-Shomron District was selected as the study setting for three strategic reasons: (1) it represents the second-largest district within CHS, encompassing both central metropolitan areas with major tertiary medical centers and peripheral rural communities; (2) the district serves diverse populations including Jewish and Arab residents, individuals across socioeconomic strata, secular and Ultra-Orthodox patients, and cultural minorities including immigrants from former Soviet Union and Ethiopia; and (3) established research collaborations facilitated data access subject to institutional ethics approval.

### Data Source and Population

#### Data Extraction and Management

Data were retrieved from CHS using the Clalit Research Data sharing platform powered by MDClone [72] for the Sharon-Shomron District, which includes populations from representative subregions and according to SES, ethnicity (Arab, Jewish-orthodox, and Jewish-general), and area of residence (central or remote according to geographic information system location data, and urban or rural). Our sample consisted of health records of all insured patients in the Sharon-Shomron District who met the inclusion criteria (“users of telehealth services”) and those who did not meet such criteria (“nonusers”).

Data included five characteristics: (1) sociodemographic characteristics (age, sex, demographic sector, area level SES, residency, and periphery types), (2) number and types of chronic health conditions, overall morbidity burden score (Charlson Comorbidity Index), (3) telehealth services usage (number of telehealth service visits per period for each service type), (4) regular (in-person) family physician services usage (number of visits per period), and (5) telehealth and regular family physician consultation outcomes: related medical diagnosis and care received post-visit (emergency room or hospitalization within 7 days).

The databases integrate electronic health records, insurance claims, demographic information, and geographic data. The database captures all health care encounters, including traditional in-person visits and telehealth consultations, providing comprehensive longitudinal health information for all enrolled members.

#### Study Population Definition

The target population comprised all CHS members aged 25 years and older residing in the Sharon-Shomron District. We established a baseline population of adults who maintained continuous membership throughout the entire study observation period.

### Eligibility Criteria and Final Study Cohort

The inclusion and exclusion criteria are listed in Textbox 1.

Textbox 1. **Inclusion and exclusion criteria.**

**Inclusion criteria**

- Active Clalit Health Services membership in Sharon-Shomron District.
- Age ≥25 years at study initiation (March 1, 2019).
- Continuous enrollment throughout the entire study period (March 1, 2019-February 28, 2022).
- Complete demographic and administrative data available.

**Exclusion criteria**

- Membership gaps or termination during the study period.
- Incomplete demographic or geographic classification data.
- Age <25 years (to focus on adult health care usage patterns).

After applying inclusion and exclusion criteria, the final analytical cohort comprised 499,607 adult members with complete data across all study variables.

### Temporal Analysis Framework

We chose the years 2019‐2021 since they represent an era where there has been an increase in the use of telehealth as a result of COVID-19 worldwide, in Israel, and in Clalit Health care services. This timeframe allows us to consider a substantial number of medical files of patients who have used telehealth in CHS.

#### Study Period Division

We designed a 3-period temporal analysis framework to capture telehealth usage patterns in relation to the COVID-19 pandemic:

1. Pre-COVID-19 period: March 1, 2019-February 28, 2020 (baseline telehealth patterns).
2. COVID-19 period: March 1, 2020-February 28, 2021 (acute pandemic response).
3. Post-COVID-19 period: March 1, 2021-February 28, 2022 (sustained adoption patterns).

This framework allows examination of telehealth adoption trajectories, identification of pandemic-specific effects, and assessment of sustained behavioral changes in health care seeking patterns.

#### Temporal Trend Analysis

For each time period, we calculated (1) overall telehealth usage rates by demographic subgroups, (2) service-specific usage patterns (telephone vs video vs TYTO services), (3) after-hours consultation frequency and timing, and (4) longitudinal changes in usage patterns across the 3 periods.

This study represents an analysis of telehealth usage patterns across 3 distinct time periods rather than longitudinal tracking of individual changes over time. Our design examines usage rates within each period among the eligible population during that specific timeframe, not repeated measures of identical individuals across periods. Each time period (pre-COVID-19, COVID-19, and post-COVID-19) includes all CHS members who met the inclusion criteria during that respective timeframe, regardless of their membership status in other periods. This cross-sectional approach to each time period allows for examination of population-level telehealth adoption patterns and demographic disparities within the context of the COVID-19 pandemic timeline, but does not permit analysis of individual-level behavioral changes or temporal interaction effects that would require following the same cohort longitudinally. The temporal framework serves to understand how telehealth usage evolved across different population segments during distinct phases of the pandemic rather than to track changes in disparity magnitude among identical individuals over time.

### Variable Definitions and Measurements

#### Primary Outcome Variables

In telehealth usage, we focused on the data obtained for “telemedicine users,” that is, those with at least 2 telemedicine visits per year, in contrast to “nonusers”—those with less than 2 telemedicine visits annually. We also examined a binary indicator defined as ≥1 telehealth consultation during each study period for broader usage analysis.

By “use of telehealth services,” we refer to 3 types of services representing different levels of involvement of patients and health care providers mentioned below.

#### Telehealth Service Categories

The telehealth service categories are listed below:

1. Telephone or video conference visits or consultations with the treating physician: voice-only or real-time video-based consultations with the patient’s regular health care provider during standard clinic hours.
2. Telephone or videoconference visits or consultations with off-hour online (“after-hours”) physician services: remote consultations available during evenings, nights, weekends, and holidays when regular clinic services are unavailable.
3. Virtual conversations through the use of “TYTO”: a small device aimed at performing 8 types of medical tests through which a health care provider can supply their rapid diagnosis. This device was reported to outperform the stand-alone digital stethoscope and otoscope and was better able to provide usable data to support a clinical encounter [73]. The TYTO Care system enables remote diagnostic consultations using integrated medical devices, including a digital stethoscope, otoscope, thermometer, blood pressure monitor, pulse oximeter, electrocardiogram, dermatoscope, and general camera.

In after-hours usage, telehealth consultations occurring outside standard clinic operating hours (evenings, nights, weekends, and holidays), calculated as a percentage of total telehealth visits. Exploring the use of telehealth consultations during after-work hours is crucial for understanding its role in enhancing health care accessibility and equity. Specifically, it is important to examine the usage trends, for example, how the demand for after-work hours telehealth services compares to traditional primary care physician visits during the same timeframe. The examination of the impact on health care outcomes is also of high importance, for example, exploring whether after-work-hours telehealth usage reduces strain on emergency departments or facilitates timely medical interventions.

#### Sociodemographic Variables

Age is categorized into 5 groups (25‐35, 35‐50, 50‐60, 60‐70, ≥70 years) based on the age at study initiation, and sex has a binary classification (male and female) based on administrative records. The demographic sector has a four-category classification reflecting Israel’s diverse population:

1. General Jewish (secular and traditional Jewish populations)
2. Arab and Bedouin (Arabic-speaking populations)
3. Religious Jewish (Ultra-Orthodox Jewish communities)
4. Druze and Cherkess (minority ethnic-religious communities)

The SES has a 3-level classification (low, medium, and high) based on Israel Central Bureau of Statistics neighborhood-level socioeconomic indices, incorporating income, education, employment, and housing quality indicators.

Residency type has a six-category classification based on community characteristics:

1. Non-Jewish settlement
2. Kibbutz (collective agricultural communities)
3. Moshav or Kfar (small agricultural communities with private farms)
4. Moatza or Ayara (regional councils and local authorities)
5. Small town
6. Large town

The periphery is classified into a five-level geographic classification based on distance from major urban centers and service availability:

1. Very peripheral
2. Peripheral
3. Medium peripheral
4. Central
5. Very central

#### Health Status Variables

##### Charlson Comorbidity Index

Validated measure of comorbidity burden incorporated 16 chronic conditions weighted by their association with mortality risk. Individual conditions included diabetes mellitus, hemiplegia or paraplegia, leukemia, lymphoma, AIDS or HIV, cerebrovascular disease, chronic pulmonary disease, congestive heart failure, dementia, liver disease, malignancy, myocardial infarction, peptic ulcer disease, peripheral vascular disease, renal disease, and rheumatic or connective tissue disease.

##### Individual Chronic Conditions

Binary indicators for each of the 16 Charlson Comorbidity Index components were extracted from diagnostic codes in electronic health records. Variable definitions are enclosed in Multimedia Appendix 1.

### Statistical Analysis Methods

#### Descriptive Analysis

We used descriptive statistics to describe the sociodemographic and health-related characteristics of the target population, types of telehealth services used, and their frequency and consultation outcomes.

We calculated frequencies and percentages for categorical variables and means with SDs for continuous variables. Telehealth usage rates were computed for each demographic subgroup across all 3 time periods, with 95% CIs calculated using exact binomial methods.

#### Temporal Trend Analysis

We assessed changes in telehealth usage across the three study periods using:

1. Chi-square tests for trend to evaluate linear changes over time.
2. Interrupted time series analysis to assess pandemic impact.
3. Calculation of rate ratios comparing the COVID-19 and post-COVID-19 periods to the pre-COVID-19 baseline.

#### Univariate Analysis

We have conducted univariate analyses to examine the relationships between sociodemographic and health-related characteristics and the various types of telehealth service use during each study period.

We examined bivariate associations between each sociodemographic factor and telehealth usage using chi-square tests of independence for categorical variables and *t* tests for continuous variables. Statistical significance was set at *P*<.05 for all analyses.

#### Multivariate Analysis

We constructed multivariable logistic regression models to assess independent associations between sociodemographic factors and telehealth usage while controlling for potential confounders. The models included all sociodemographic variables and the Charlson Comorbidity Index as predictors.

A multivariable logistic regression model was constructed to assess the independent association of each sociodemographic and health-related factor with the likelihood of telehealth usage. Bootstrapping (1000 resamples) was applied to ensure robust estimates. The adjusted bootstrapped odds ratios (ORs) and their 95% CIs were reported to quantify the strength and direction of associations. The model controlled for confounding by including all predictors simultaneously. Statistical significance was set at *P*<.05. Model fit was assessed using the Hosmer-Lemeshow goodness-of-fit test. Multicollinearity was examined using the variance inflation factor to ensure no strong correlations between independent variables.

#### Model Specification

The model specification is classified into three categories:

1. Outcome: binary telehealth usage (≥1 consultation per period).
2. Predictors: age group, sex, demographic sector, SES, residency type, periphery classification, Charlson Comorbidity Index
3. Reference categories: age 25‐35 years, female sex, General Jewish sector, high SES, large town residency, very central periphery

For robust estimation, we applied bootstrapping with 1000 resamples to obtain robust SEs and 95% CIs, accounting for potential nonnormal distribution of residuals and heteroscedasticity.

#### Model Diagnostics

The model diagnostics are classified into three categories: (1) Hosmer-Lemeshow goodness-of-fit test to assess model calibration, (2) variance inflation factor analysis to detect multicollinearity (variance inflation factor>10 considered problematic), and (3) residual analysis to identify influential observations.

#### After-Hours Analysis

We conducted specialized analyses of after-hours telehealth usage, including (1) calculation of after-hours usage percentages by demographic subgroups and time periods, (2) chi-square tests to assess differences in after-hours usage patterns, and (3) logistic regression models predicting after-hours versus regular-hours telehealth use.

#### Missing Data Management

We assessed patterns of missing data across all variables. Given the administrative nature of the data source, missing data rates were minimal (<1% for most variables). Complete case analysis was performed for the primary analyses, with sensitivity analyses conducted using multiple imputation for variables with >1% missing data.

#### Software and Reproducibility

All analyses were conducted using R version 4.0 or later (R Core Team). Statistical code and analysis protocols are available upon request to ensure reproducibility.

### Ethical Considerations

This study was approved by the CHS Institutional Review Board (IRB number 0085-22-COM). Given the retrospective nature of using deidentified administrative data, informed consent was waived. All analyses adhered to HIPAA (Health Insurance Portability and Accountability Act) privacy standards and institutional data use agreements. Patient confidentiality was maintained throughout the research process, with no individual-level identifiers retained in the analytical dataset.

## Results

### Cohort Characteristics

Our study population comprised 499,607 adult Clalit Healthcare Services members (aged ≥25 years) in the Sharon-Shomron district with continuous membership throughout the study period (from March 1, 2019, to February 28, 2021).

The population was 51.7% (258,471/499,607) female with a mean age of 50.6 (SD 16.5) years at baseline. The majority (413,940/499,607, 82.9%) belonged to the General Jewish demographic sector, while 15.4% (76,895/499,607) were from the Arab and Bedouin sector. Most participants (271,442/499,607, 54.3%) were classified as having medium SES, and the majority resided in small (187,315/499,607, 37.5%) or large towns (124,157/499,607, 24.8%) within central (170,734/499,607, 34.2%) or very central (186,121/499,607, 37.3%) areas.

Parameters of sociodemographic variables of the population are summarized in Table 1. The population exhibited a relatively low mean Charlson Comorbidity Index of 2.0 (SD 2.5), suggesting a relatively healthy cohort.

**Table 1.** Sociodemographic characteristics of the population.

Variables	Values, n (%)
Sex	
Female	258,471 (51.7)
Male	241,136 (48.3)
Age group (years)	
25‐35	102,533 (20.5)
35‐50	170,465 (34.1)
50‐60	75,233 (15.1)
60‐70	79,096 (15.8)
70+	72,280 (14.5)
Demographic sector	
General Jewish	413,940 (82.9)
Arab or Bedouin	76,895 (15.4)
Religious Jewish	7630 (1.5)
Druze or Cherkess	1142 (0.2)
SES^a^ 3-level scale	
Low	56,154 (11.2)
Medium	271,442 (54.3)
High	172,011 (34.4)
Residency type	
Non-Jewish settlement	73,969 (15.8)
Kibbutz	21,402 (4.3)
Moshav or Kfar	39,364 (8.9)
Moatza or Ayara	53,400 (11.7)
Small town	187,315 (37.5)
Large town	124,157 (25.9)
Periphery type	
Very peripheral	10,259 (2.1)
Peripheral	14,173 (3.8)
Medium peripheral	118,320 (24.7)
Central	170,734 (34.2)
Very central	186,121 (37.3)

a SES: socioeconomic status.

The most prevalent chronic conditions were diabetes mellitus (88,350/499,607, 17.7%), chronic pulmonary disease (75,726/499,607, 15.2%), and cerebrovascular disease (46,952/499,607, 9.4%). The distribution of the chronic health conditions in the population is summarized in Table 2.

**Table 2.** Chronic health conditions of the population.

Characteristics	Values (N=499,607), n (%)
Diabetes mellitus	88,350 (17.7)
Hemiplegia or paraplegia	8821 (1.8)
Leukemia	1523 (0.3)
Lymphoma	5040 (1.0)
AIDS or HIV	637 (0.1)
Cerebrovascular disease	46,952 (9.4)
Chronic pulmonary disease	75,726 (15.2)
Congestive heart failure	17,011 (3.4)
Dementia	18,806 (3.8)
Liver disease	41,931 (8.4)
Malignancy	36,393 (7.3)
Myocardial infarction	20,165 (4.0)
Peptic ulcer disease	23,498 (4.7)
Peripheral vascular disease	14,004 (2.8)
Renal disease	24,900 (5.0)
Rheumatic or connective tissue disease	20,257 (4.1)

The population exhibited a mean Charlson Comorbidity Index score of 2.0 (SD 2.5), suggesting a relatively healthy cohort, which we will include in the table format.

### Telehealth Usage Patterns

#### Temporal Trends in Telehealth Adoption

Telehealth usage demonstrated a marked increase during the study period. In the pre-COVID-19 period (March 2019-February 2020), 4% (20,146/499,607) of members used at least 1 telehealth service. This proportion more than doubled to 8.9% (44,678/499,607) during the COVID-19 period (March 2020-February 2021) and further increased slightly to 9.4% (46,868/499,607) in the post-COVID-19 period (March 2021-February 2022).

Telephone services constituted the primary mode of telehealth delivery across all periods, with usage increasing from 4% (20,146/499,607) pre-COVID-19 to 8.7% (43,494/499,607) during COVID-19, and 9.1% (45,225/499,607) post-COVID-19. TYTO online services, while less frequently used, exhibited proportionally greater growth, with usage rates increasing from 0.04% (178/499,607) pre-COVID-19 to 0.4% (1787/499,607) during COVID-19 and 0.5% (2363/499,607) post-COVID-19.

The telehealth service usage in the population is summarized in Table 3.

**Table 3.** Telehealth service usage during the 3 study periods.

Type of telehealth usage	Study period		
	Pre-COVID-19 period, n (%)	COVID-19 period, n (%)	Post-COVID-19 period, n (%)
TYTO online services user	178 (0.04)	1787 (0.4)	2363 (0.5)
Telephone online services user	20,146 (4)	43,494 (8.7)	45,225 (9.1)
Any online services user	20,264 (4.1)	44,678 (8.9)	46,868 (9.4)

#### Sociodemographic Determinants of Telehealth Usage

Significant disparities in telehealth usage were observed across all sociodemographic variables (*P*<.01 for all comparisons), with these differences persisting across all study periods.

The distribution of telehealth service usage by different sociodemographic factors of the population during each study period is summarized in Table 4.

**Table 4.** Telehealth service usage by sociodemographic factors by study period.

Categories and subcategory		Total sample, n	Pre-COVID period, n (%)	COVID period, n (%)	Post-COVID period, n (%)
Sex					
Female		258,471	11,985 (4.6)	24,581 (9.5)	26,702 (10.3)
Male		241,136	8279 (3.4)	20,097 (8.3)	20,166 (8.4)
Age group (years)					
25‐35		102,533	8059 (7.9)	16,749 (16.3)	18,333 (17.9)
35‐50		170,465	6415 (3.8)	14,379 (8.4)	15,187 (8.9)
50‐60		75,233	1875 (2.5)	4593 (6.1)	4475 (5.9)
60‐70		79,096	2044 (2.6)	4820 (6.1)	4744 (6.0)
70+		72,280	1871 (2.6)	4137 (5.7)	4129 (5.7)
Demographic sector					
General Jewish		413,940	19,366 (4.7)	42,464 (10.3)	44,796 (10.8)
Arab or Bedouin		76,895	438 (0.6)	1214 (1.6)	1125 (1.5)
Religious Jewish		7630	443 (5.8)	966 (12.7)	904 (11.8)
Druze or Cherkess		1142	17 (1.5)	32 (2.8)	43 (3.8)
SES^a^ −3 level scale					
Low		56,154	587 (1.0)	1404 (2.5)	1328 (2.4)
Medium		271,442	11,421 (4.2)	25,123 (9.3)	26,476 (9.8)
High		172,011	8256 (4.8)	18,151 (10.6)	19,064 (11.1)
Residency type					
Non-Jewish settlement		73,969	371 (0.5)	1072 (1.4)	993 (1.3)
Kibbutz		21,402	680 (3.2)	1468 (6.9)	1730 (8.1)
Moshav or Kfar		39,364	1743 (4.4)	3726 (9.5)	3901 (9.9)
Moatza or Ayara		53,400	2437 (4.6)	5118 (9.6)	5561 (10.4)
Small town		187,315	8272 (4.4)	18,770 (10.0)	19,838 (10.6)
Large town		124,157	6761 (5.4)	14,524 (11.7)	14,845 (12.0)
Periphery type					
Very peripheral		10,259	466 (4.5)	980 (9.6)	1115 (10.9)
Peripheral		14,173	551 (3.9)	1163 (8.2)	1279 (9.0)
Medium peripheral		118,320	3500 (3.0)	7576 (6.4)	8138 (6.9)
Central		170,734	6049 (3.5)	13,786 (8.1)	14,350 (8.4)
Very central		186,121	9698 (5.2)	21,173 (11.4)	21,986 (11.8)

a SES: socioeconomic status.

#### Gender Differences

Women consistently demonstrated higher telehealth adoption rates than men across all periods. These rates are listed as follows:

1. Pre-COVID-19: females (11,985/258,471, 4.6%) versus males (8279/241,136, 3.4%); 35% higher usage by females.
2. COVID-19: females (24,581/258,471, 9.5%) versus males (20,097/241,136, 8.3%); 14% higher usage by females.
3. Post-COVID-19: females (26,702/258,471, 10.3%) versus males (20,166/241,136, 8.4%); 23% higher usage by females.

This gender disparity was statistically significant (*P*<.01) and persisted throughout all study periods.

#### Age-Related Patterns

A pronounced inverse relationship was observed between age and telehealth usage. The youngest age group (25‐35 years) exhibited the highest usage rates (pre-COVID-19: 8059/102,533, 7.9%; COVID-19: 16,749/102,533, 16.3%; post-COVID-19: 18,333/102,533, 17.9%), while the oldest age group (≥70 years) demonstrated the lowest rates (pre-COVID-19: 1871/72,280, 2.6%; COVID-19: 4137/72,280, 5.7%; post-COVID-19: 4129/72,280, 5.7%; *P*<.01).

The youngest adults (25-35 years) consistently used telehealth at nearly triple the rate of older adults (70+ years). This pattern persisted across all time periods, with the difference remaining statistically significant (*P*<.01). Notably, while both groups showed substantial increases in adoption from pre- to post-COVID-19 periods, the absolute gap in usage (between those groups) widened from 5.3 percentage points to 12.2 percentage points.

#### Ethnocultural Variations

Substantial disparities in telehealth usage were observed across ethnocultural sectors. The Religious Jewish sector exhibited the highest usage rates (pre-COVID-19: 443/7630, 5.8%; COVID-19: 966/7630, 12.7%; post-COVID-19: 904/7630, 11.8%), while the Arab and Bedouin sector demonstrated markedly lower rates than the general Jewish population (pre-COVID-19: 438/76,895, 0.6%; COVID-19: 1214/76,895, 1.6%; post-COVID-19: 1125/76,895, 1.5%; *P*<.01).

The nearly 10-fold difference between the highest-adopting sector (Religious Jewish) and the lowest-adopting sector (Arab and Bedouin) represents one of the most pronounced disparities in the study. Each demographic sector category proportion differed significantly from the others at the 0.05 level during each study period, suggesting deep-rooted cultural, linguistic, technological, or structural barriers to telehealth adoption in certain communities.

#### Socioeconomic Gradient

The data revealed one of the most profound disparities in telehealth adoption across socioeconomic lines: a strong positive association was observed between SES and telehealth usage. Members with high SES used telehealth services at nearly 4 times the rate of those with low SES (pre-COVID-19: 8256/172,011, 4.8% vs 587/56,154, 1.0%; COVID-19: 18,151/172,011, 10.6% vs 1404/56,154, 2.5%; post-COVID-19: 19,064/172,011, 11.1% vs 1328/56,154, 2.4%; *P*<.01).

This nearly 5-fold difference between high and low SES groups represents a significant digital divide with important health equity implications. While both groups saw increases in adoption during the COVID-19 pandemic, the proportional gap remained relatively unchanged across the periods, suggesting that the pandemic did not meaningfully close the socioeconomic telehealth divide.

#### Geographic Variations

Telehealth usage varied significantly by residence type and peripherality. Members residing in large towns demonstrated the highest usage rates (pre-COVID-19: 6761/124,157, 5.4%; COVID-19: 14,524/124,157, 11.7%; post-COVID-19: 14,845/124,157, 12%), while those in non-Jewish settlements exhibited the lowest rates (pre-COVID-19: 371/73,969, 0.5%; COVID-19: 1072/73,969, 1.4%; post-COVID-19: 993/73,969, 1.3%; *P*<.01).

The data show more than a 10-fold difference in telehealth adoption between large towns and non-Jewish settlements. There were no significant differences in usage between patients residing in a Kibbutz (a collective community in Israel, traditionally based on agriculture, where members share ownership, resources, and responsibilities), a Moshav (small agricultural community with personally owned household farms), or small towns, suggesting that the urban-rural divide may be less pronounced than cultural or community-specific factors.

**Figure 1.**
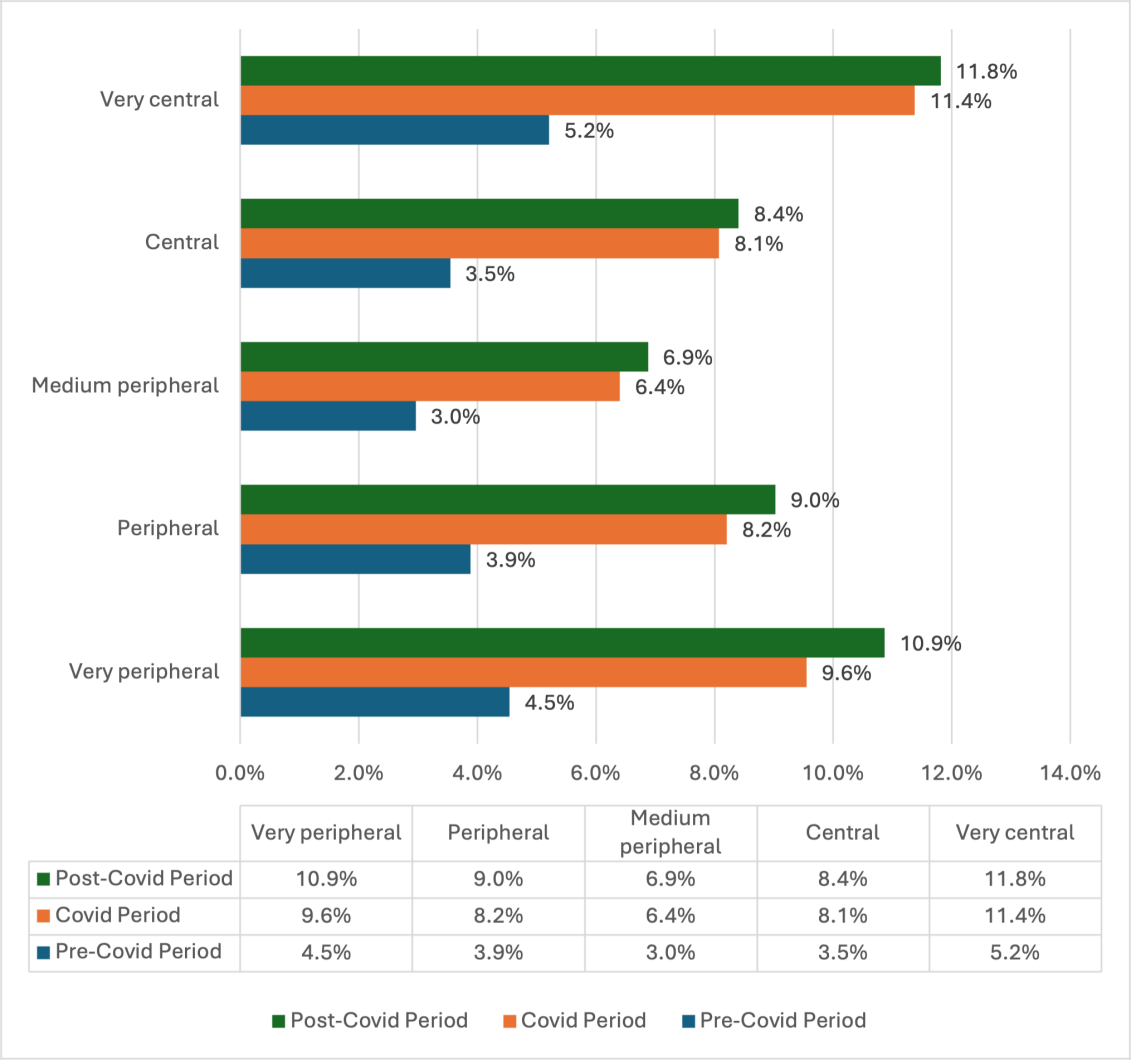
Percentage of patients who used any telehealth services by periphery type and study period.

Notably, a U-shaped relationship was observed with peripherality, with both very peripheral and very central locations demonstrating higher usage rates (pre-COVID-19: 466/10,259, 4.5% to 9698/186,121, 5.2%; COVID-19: 980/10,259, 9.6% to 21,173/186,121, 11.4%; post-COVID-19: 1115/10,259, 10.9% to 21,986/186,121, 11.8%) compared to medium peripheral locations (pre-COVID-19: 3500/186,121, 3%; COVID-19: 7576/118,320, 6.4%; post-COVID-19: 1115/10,259, 6.9%; *P*<.01) (Figure 1).

There were no significant differences in the percentage of telehealth services usage between patients who reside in locations of peripheral and central types, as well as between patients who reside in locations of peripheral and very peripheral types.

This U-shaped distribution challenges conventional assumptions about telehealth adoption. Both very peripheral and very central locations showed higher adoption rates than medium peripheral areas. This might reflect the different motivations driving telehealth adoption: in very peripheral areas, telehealth may overcome physical distance barriers, while in very central areas, it may cater to tech-savvy populations seeking convenience.

#### Intersectionality of Sociodemographic Determinants

We used a multivariable logistic regression model to identify sociodemographic and health-related predictors of telehealth usage across 3 periods: pre-COVID-19, COVID-19, and post-COVID-19. Telehealth use, defined as at least 1 consultation during the study period, served as the primary binary outcome. Bootstrapping (1000 resamples) was applied for robust estimates, with adjusted odds ratios (ORs) and 95% CIs reported (Table 5). All predictors were included simultaneously to control for confounding, and statistical significance was set at *P*<.05.

**Table 5.** Sociodemographic factors of telehealth services usage during any period and results of multivariable logistic regression.

Factor	Exp (B)	95% CI	*P* value^a^
Sex (male)	0.802	0.790-0.814	<.001
Age group (years)			
35‐50 (vs 25‐35)	0.513	0.504-0.523	<.001
50+ (vs 25‐35)	0.290	0.283-0.298	<.001
Sector			
Arab or Bedouin or Druze (vs General Jewish)	0.183	0.175-0.192	<.001
Ultra-Religious Jewish (vs General Jewish)	1.215	1.150-1.284	<.001
Periphery type			
Peripheral (vs very central)	0.824	0.795-0.854	<.001
Medium peripheral (vs very central)	0.806	0.789-0.824	<.001
Central (vs very central)	0.816	0.802-0.830	<.001
Charlson Comorbidity Index (pre-COVID-19 period)	1.023	1.018-1.028	<.001
SES^b^			
Medium	1.314	1.254-1.378	<.001
High	1.353	1.289-1.421	<.001
Constant	0.484	—^c^	<.001

a Bootstrapped.

b SES: socioeconomic status.

c Not applicable.

The results reveal notable disparities in telehealth usage across demographic and geographic groups. Controlling for all included covariates, men were 20% less likely to use telehealth services compared to women (OR 0.802, 95% CI 0.790‐0.814).

Age also played a significant role: individuals aged 35‐50 years were 49% less likely to use telehealth than those aged 25‐35 years (OR 0.513, 95% CI 0.504‐0.523), and those aged 50+ years were 71% less likely (OR 0.290, 95% CI 0.283‐0.298), suggesting that younger adults, more tech-savvy and with fewer barriers, are the primary adopters, beyond all other characteristics.

Even after controlling for other personal and community-related factors, ethnic and cultural sectors still exhibited stark contrasts. Individuals from the Arab, Bedouin, and Druze sectors were 82% less likely to use telehealth services (OR 0.183, 95% CI 0.150‐0.284), indicating substantial cultural or systemic barriers. Conversely, members of the Ultra-Orthodox Jewish sector were 21% more likely to use telehealth than the General Jewish population (OR 1.215, 95% CI 1.150‐1.284), possibly due to tailored community solutions or preferences for remote care.

Geography further influenced usage, beyond ethnicity: residents of peripheral (OR 0.824, 95% CI 0.795-0.854), medium peripheral (OR 0.806, 95% CI 0.789-0.824), and even central areas (OR0.816, 95% CI 0.802-0.830) were all significantly less likely to use telehealth compared to those living in very central regions, where access to infrastructure and services is likely better.

A higher Charlson Comorbidity Index is associated with a 2.3% increase in the likelihood of using telehealth services per unit increase in the index (OR 1.023, 95% CI 1.018‐1.028). This highlights the role of telehealth in managing patients with chronic or complex conditions.

People in medium and high SES (OR 1.314, 95% CI 1.254-1.378 and OR 1.353, 95% CI 1.289-1.421, respectively) were significantly more likely to use telehealth services, as compared to people with low SES.

### After-Hours Telehealth Usage Analysis—Key Findings

#### Temporal Distribution: Shifting Patterns of Usage

Our analysis reveals significant shifts in after-hours telehealth usage patterns across different time periods and demographic groups, highlighting important trends in health care accessibility.

Our analysis reveals significant shifts in after-hours telehealth usage patterns across different time periods and demographic groups, highlighting important trends in health care accessibility.

A notable shift occurred in the temporal distribution of telehealth consultations across the study periods: the percentage of telehealth consultations occurring during after-work hours has steadily decreased from the pre-COVID-19 era through the postpandemic period. Before COVID-19, after-hours telehealth consultations represented 65% (324,744/499,607) of total telehealth usage, indicating that telehealth primarily served as an after-hours alternative to traditional care. During the pandemic, this figure dropped to approximately 55% and further declined to 49% in the post-COVID-19 period.

Notable changes are listed as follows:

1. Pre-COVID-19 period: the highest percentage of after-hours consultations.
2. COVID-19 period: 10% decrease in after-hours consultations compared to pre-COVID-19.
3. Post-COVID-19 period: further decline to 49% of pre-COVID-19 after-hours consultation levels.

This decline suggests a fundamental transformation in telehealth’s role within the health care ecosystem, evolving from a predominantly after-hours convenience to an integrated service available throughout the day.

#### Sociodemographic Determinants of After-Hours Telehealth Usage

##### Gender Patterns Show Subtle Shifts

Both females and males equally relied on after-work hours telehealth consultations during the pre-COVID-19 period (65% for both sexes; 10,004/15,344 for females and 6607/10,108 for males). This symmetry may reflect similar barriers to accessing care during regular hours before the widespread adoption of telehealth during the COVID-19 pandemic.

A slight decline in the percentage of after-work hours consultations occurred during the COVID-19 period for both sexes, but males showed a marginally higher percentage compared to females (1% difference). This suggests that males might have slightly shifted their usage preferences or schedules during the pandemic.

The percentage continued to decline in the post-COVID-19 period, with females showing a slightly higher percentage than males (2% difference). This indicates that while overall reliance on after-work hours decreased for both sexes, females remained somewhat more likely to use telehealth services during these times compared to males.

In conclusion, gender differences in after-hours telehealth usage were minimal but noteworthy:

1. Before the pandemic, usage was identical between males and females (65%).
2. During the COVID-19 pandemic, males showed slightly higher after-hours usage (approximately 1% difference).
3. Postpandemic, the pattern reversed with females demonstrating modestly higher usage (approximately 2% difference).

##### Socioeconomic Disparities Persist

Our findings reveal consistent socioeconomic gradients in after-hours telehealth usage across all time periods (Table 6). The percentages in the table represent the number of after-hours visits out of the total number of visits from all online encounters.

**Table 6.** After-hours telehealth usage by demographic characteristics across study periods.

Characteristics	Pre-COVID-19, n/N (%)	COVID-19, n/N (%)	Post-COVID-19, n/N (%)	*P* value^a^
Sex				
Female	10,004/15,344 (65)	18,532/34,031 (54)	20,041/39,931 (50)	<.001
Male	6607/10,108 (65)	14,869/27,124 (55)	13,673/28,247 (48)	<.001
Socioeconomic status				
Low	539/794 (68)	1153/1980 (58)	1095/2069 (53)	<.001
Medium	9636/14,616 (66)	19,159/34,795 (55)	19,379/38,780 (50)	<.001
High	6436/10,114 (64)	13,089/24,380 (54)	13,240/27,329 (48)	<.001
Demographic sector				
General Jewish	15,866/24,403 (65)	31,729/58,243 (54)	32,240/65,329 (49)	<.001
Arab or Bedouin	333/521 (64)	813/1471 (55)	695/1449 (48)	<.001
Religious Jewish	402/581 (69)	838/1401 (60)	750/1347 (56)	<.001
Druze or Cherkess	10/19 (53)	21/40 (53)	29/53 (55)	.34
Age group (years)				
25‐35	6485/10,261 (63)	12,825/24,387 (53)	13,907/28,827 (48)	<.001
35‐50	5339/8070 (66)	10,730/19,221 (56)	10,711/21,481 (50)	<.001
50‐60	1558/2344 (66)	3436/6056 (57)	3152/6106 (52)	<.001
60‐70	1640/2454 (67)	3518/6206 (57)	3162/6292 (50)	<.001
70+	1589/2395 (66)	2892/5285 (55)	2782/5472 (51)	<.001
Residency type				
Non-Jewish settlement	291/444 (66)	708/1286 (55)	597/1258 (47)	<.001
Kibbutz	512/809 (63)	933/1851 (50)	988/2279 (43)	<.001
Moshav or Kfar	1326/2055 (65)	2646/4903 (54)	2580/5339 (48)	<.001
Moatza or Ayara	1945/3008 (65)	3745/6749 (55)	3862/7906 (49)	<.001
Small town	6645/10,280 (65)	13,923/25,560 (54)	14,258/28,857 (49)	<.001
Large town	5892/8928 (66)	11,446/20,806 (55)	11,429/22,539 (51)	<.001
Periphery type				
Very peripheral	367/580 (63)	740/1341 (55)	776/1675 (46)	<.001
Peripheral	452/711 (64)	909/1643 (55)	921/1852 (50)	<.001
Medium peripheral	2865/4284 (67)	5620/10,197 (55)	5604/11,570 (48)	<.001
Central	4980/7647 (65)	10,240/18,607 (55)	10,222/20,625 (50)	<.001
Very central	7947/12,302 (65)	15,892/29,367 (54)	16,191/32,456 (50)	<.001

a *P* values from the chi-square test for trend across the three time periods.

1. Low SES individuals maintained the highest percentage of after-hours telehealth consultations (pre-COVID-19: 539/794, 68%; COVID-19: 1153/1980, 58%; post-COVID-19: 1095/2069, 53%).
2. Medium SES groups showed intermediate usage (pre-COVID-19: 9636/14,616, 66%; COVID-19: 19,159/34,795, 55%; post-COVID-19: 19,379/38,780, 50%).
3. High SES populations demonstrated the lowest after-hours dependency (pre-COVID-19: 6436/10,114, 64%; COVID-19: 13,089/24,380, 54%; post-COVID-19: 13,240/27,329, 48%).

The higher reliance on after-work hours by low SES individuals during the pre-COVID-19 period highlights socioeconomic disparities in health care access. While the gap between socioeconomic groups narrowed slightly during the pandemic (when telehealth became more mainstream and when people remained at home), these persistent differences highlight ongoing barriers to daytime health care access for disadvantaged populations.

##### Cultural Factors Influence Usage Patterns

During the pre-COVID-19 period, the religious Jewish sector (402/581, 69%) had the highest percentage of after-work hours telehealth consultations, followed by General Jewish (15,866/24,403, 65%), Arab or Bedouin (333/521, 64%), and Druze or Cherkess (10/19, 53%) (Table 6). The higher usage among the Religious Jewish sector might be due to cultural or lifestyle factors, such as working hours or religious commitments during the day, driving reliance on after-work hours services.

The percentages declined across all sectors during the COVID-19 period. The Religious Jewish sector (838/1401, 60%) still had the highest percentage, followed by Arab or Bedouin (813/1471, 55%) and General Jewish (31,729/58,243, 54%), while Druze or Cherkess remained constant at 53% (21/40). This decline across sectors indicates a shift in usage patterns, possibly due to the increased availability of telehealth during regular hours as part of the pandemic response.

During the post-COVID-19 period, usage continued to decline overall, with the Religious Jewish sector (750/1347, 56%) maintaining the highest percentage, followed by Druze or Cherkess (29/53, 55%), General Jewish (32,240/65,329, 49%), and Arab or Bedouin (695/1449, 48%). Interestingly, the Druze or Cherkess sector demonstrated a rise in its percentage from the COVID-19 period (from 53% to 55%), suggesting a stabilization or increasing preference for after-work hours telehealth services in this group.

Distinct patterns emerged across different cultural and religious sectors: (1) Religious Jewish communities consistently demonstrated the highest after-hours usage (pre-COVID-19: 69%; COVID-19: 60%; and post-COVID-19: 56%). (2) General Jewish populations showed a significant decline over time (pre-COVID-19: 65%; COVID-19: 54%; and post-COVID-19: 49%). (3) Arab or Bedouin communities exhibited the steepest reduction (pre-COVID-19: 64%; COVID: 55%; and post-COVID-19: 48%). (4) Druze or Cherkess groups displayed unique stability, maintaining relatively consistent usage patterns (pre-COVID-19: 53%; COVID-19: 53%; and post-COVID-19: 55%).

##### Age-Related Usage Patterns

Our findings reveal distinctive patterns across age groups (Table 6): (1) pre-COVID-19 period: after-hours telehealth usage was relatively consistent across age groups, with older adults (60‐70 years) showing the highest usage (1640/2454, 67%), followed closely by those aged 70+ years (1589/2395, 66%). (2) COVID-19 period: all age groups experienced a decline in after-hours usage, with the 25‐ to 35-year age group showing the most pronounced reduction (10% decrease). (3) post-COVID-19 period: the 50‐ to 60-year age group maintained the highest after-hours usage (3152/6106, 52%), while the youngest adults (25-35 years) showed the lowest reliance (13,907/28,827, 48%).

##### Geographical and Residential Variations

Telehealth usage patterns varied across different residency types and geographical locations:

The residency types (Table 6) are listed as follows:

1. Pre-COVID-19: usage was remarkably consistent across residency types (63%‐66%).
2. COVID-19 period: a decline is observed across all residency types during the pandemic. Non-Jewish settlements (708/1286), large towns (11,446/20,806), and Moatza or Ayara (3745/6749) maintained higher usage (all 55%), while Kibbutz communities showed lower reliance (933/1851, 50%).
3. Post-COVID-19: after-work hours usage declined further. Large towns maintained the highest after-hours usage (11,429/22,539, 51%), while Kibbutz communities showed the lowest (988/2279, 43%), representing a 20% decrease from prepandemic levels.

The periphery status (Table 6) is listed as follows:

1. Pre-COVID-19: medium peripheral areas showed the highest after-hours usage (2865/4284, 67%), while very peripheral areas had the lowest (367/580, 63%).
2. COVID-19 period: usage declined uniformly across all periphery types to approximately 55%.
3. Post-COVID-19: a further decline is seen in the post-COVID-19 period. Peripheral (921/1852), central (10,222/20,625), and very central areas (16,191/32,456) maintained slightly higher usage (all 50%), while very peripheral areas showed the lowest reliance (776/1675, 46%).

These geographical variations suggest that telehealth adoption and usage patterns are influenced by local health care infrastructure, community characteristics, and possibly technological accessibility. The pronounced reduction in after-hours usage in rural communities (Kibbutz) may reflect better integration of telehealth into regular care hours or alternative health care solutions in these settings.

## Discussion

### Importance of the Study

The rapid integration of telehealth services into health care delivery systems has been accelerated by the COVID-19 pandemic, transforming how patients access care [73,74]. However, this digital transformation raises critical questions about equitable access across diverse populations.

The data presented from our study offers valuable insights into telehealth usage patterns among nearly half a million adult members in the Sharon-Shomron district of CHS, the largest Israeli insurer and provider of health services. This discussion examines the patterns and their implications through an equity lens, exploring how various sociodemographic factors intersect to create disparities in digital health access and potentially impact health outcomes.

### Israeli Context and Legal Framework

Israel’s National Health Insurance Law (1994) guarantees universal access to health care services for all residents, establishing the state’s legal obligation to ensure equitable care access. Within this framework, digital health disparities represent not only equity concerns but potential violations of the fundamental health care access guarantees. This legal context underscores the urgency of addressing telehealth disparities to fulfill Israel’s commitment to universal health care coverage.

Israel presents a unique case study for examining intersectional disparities in telehealth due to its diverse population and universal health care system. Research by Reges et al [69] documented significant variations in telehealth usage across different segments of Israeli society during the COVID-19 pandemic, with ethnicity as the most discriminatory predictor linked with telemedicine use. Work by Penn and Laron [70] found that among Arab Israeli communities, women older than 65 years with chronic conditions faced compounded barriers including a lack of awareness, lower digital literacy, and language barriers. Geographic disparities were documented by Mendels and Wiener [71], who found significant differences between central urban areas and peripheral communities. Socioeconomic gradients were examined by Levin-Zamir and Bertschi [75], demonstrating that digital health literacy followed clear socioeconomic patterns. During the pandemic, these pre-existing characteristics dominated, as documented by Reicher et al [76]. Research by Hoffer-Chudner et al [77] explored Ultra-Orthodox Jewish women’s attitudes, finding preparedness for adoption via dedicated “kosher” medical gadgets. Zigdon et al [78] examined organizational factors, suggesting that policies and culturally adaptive approaches significantly influenced intersectional disparities.

### Key Findings and Their Implications

#### Temporal Evolution During COVID-19

Our data demonstrates significant expansion in telehealth usage, with usage more than doubling from pre-COVID-19 (4.06%) to post-COVID-19 (9.38%) periods. This dramatic increase demonstrates how the pandemic functioned as a catalyst for telehealth adoption, with the sustained high usage in the post-COVID-19 period suggesting a fundamental shift in health care delivery preferences and behaviors rather than just a temporary response to pandemic restrictions.

Similar patterns were observed by Baum et al [79] and Patel et al [73], who noted dramatic increases followed by plateauing. The predominance of telephone services highlights the importance of familiar, low-technology solutions, aligning with Rodriguez et al [36] findings.

#### Persistent Disparities Across Demographics

Gender disparities favoring women align with Fischer et al [80] findings across multiple health care systems, potentially reflecting health care-seeking behaviors and caregiving responsibilities [36].

The pronounced age-related digital divide, with the youngest cohorts using telehealth at triple the rate of the oldest groups, reflects well-documented patterns. Haimi et al [81] reported older adults’ concerns about telemedicine quality, while Lam et al [37] found that older adults faced multiple barriers. Roberts and Mehrotra [82] documented that 26.3% of Medicare beneficiaries lacked digital access, particularly affecting older adults and communities of color. This age-related disparity is particularly concerning, given that older adults typically have higher health care needs and could potentially benefit most from the convenience of telehealth services [24,37,83].

The nearly 4-fold socioeconomic difference aligns with Eberly et al [3] research, suggesting possible ongoing access barriers and income-related disparities. Cultural variations, with Religious Jewish communities showing the highest usage and Arab and Bedouin communities showing dramatically lower rates, reflect patterns observed by Rodriguez et al [36] among minority populations and Yoon et al [84] regarding linguistic minorities.

The geographic variations in telehealth usage present an intriguing pattern. The higher rates in large towns compared to non-Jewish settlements align with expected urban-rural divides in technology access. However, the U-shaped relationship with peripherality, where both very peripheral and very central locations demonstrated higher usage than medium peripheral locations, may indicate a more complex dynamic.

This pattern may reflect several things: targeted telehealth promotion in very peripheral areas to address physical access barriers, greater technology adoption and comfort in very central areas, different health care resource allocation patterns across geographic regions, and potential variations in telehealth service design and implementation across localities.

Similar geographic complexities have been documented by Chu et al, who identified that telemedicine adoption increased in rural and remote areas during the COVID-19 pandemic, but its use increased in urban and less rural populations [85]. Drake et al further demonstrated that telehealth adoption in rural communities varied significantly based on the presence of targeted implementation support and provider training [86].

In very peripheral areas, telehealth may address physical access barriers, consistent with findings by Hirko et al that rural patients valued telehealth primarily for reducing travel burden [44]. Conversely, in very central areas (socially advantaged neighborhoods), adoption may be driven by convenience and technological readiness, as suggested by Weiner et al in their analysis of urban telehealth adoption patterns [87].

The lower adoption in medium peripheral areas and non-Jewish settlements may reflect infrastructure limitations or community-specific barriers. It may also reflect the intersection between geography, SES, and culture.

Saeed et al [88] identified reliable internet connectivity as a critical prerequisite for telehealth equity, while Patel et al [73] noted that broadband access varies substantially across communities even within the same geographical region.

#### After-Hours Usage Transformation

The findings found in this study have significant implications for health care policy and service delivery. The overall decline in after-hours telehealth usage suggests successful integration of telehealth into standard care hours, potentially improving health care system efficiency. However, the persistent socioeconomic disparities highlight the ongoing need for targeted interventions to improve health care equity.

The continued higher reliance on after-hours services among lower SES groups underscores the need for targeted interventions to ensure equitable health care access.

In the post-COVID-19 period, the 50‐ to 60-year age group maintained the highest after-hours usage (52%), while the youngest adults (25-35 years) showed the lowest reliance (48%). These patterns suggest that younger populations adapted more readily to regular-hours telehealth services during and after the pandemic, while older adults maintained a slightly higher preference for after-hours consultations, possibly due to greater healthcare needs or established usage patterns.

As health care systems continue to evolve postpandemic, these findings emphasize the importance of maintaining flexible telehealth scheduling options, particularly for vulnerable populations who rely more heavily on after-hours services. Additionally, the observed cultural and religious variations suggest that telehealth implementation strategies should be tailored to meet the specific needs of diverse communities.

The significant shift in after-hours telehealth usage patterns represents a fundamental transformation in telehealth’s function within the health care ecosystem. Prepandemic, telehealth primarily served as an alternative access point outside regular hours, aligning with Mehrotra’s characterization of telehealth as a convenience-oriented service, emphasizing that before the pandemic, telemedicine was mostly used by patients in remote and rural areas of Australia, Canada, and the United States to videoconference with specialists [89].

During and after the pandemic, it evolved into an integrated component of routine health care delivery throughout the day. This transformation reflects what Wosik et al described as the “mainstreaming” of telehealth—its evolution from a niche service to a core health care delivery channel [90]. The normalization of telehealth during regular hours suggests what Dorsey and Topol (2020) called a “virtualist” approach to care, where digital interactions become standard rather than exceptional [1].

The implications of this shift extend beyond mere scheduling flexibility. As Keesara et al argued, the integration of telehealth into routine care represents a catalyst for broader digital transformation in health care delivery models [91]. This mainstreaming may facilitate what Bokolo termed “hybrid care models”—integrated approaches that strategically blend in-person and virtual care based on clinical appropriateness rather than merely emergency conditions [92].

#### Intersectionality and Compounded Barriers

Our findings show that, in general, older individuals, males, and residents of peripheral areas are less likely to use telehealth, highlighting a need for targeted outreach and education to improve adoption in these groups. Significant underutilization by Arab, Bedouin, or Druze populations suggests the need for culturally sensitive strategies to improve telehealth accessibility and trust within these communities. Lower usage rates in peripheral and medium peripheral areas may reflect geographic inequities. Investment in digital infrastructure and incentives for telehealth adoption in these regions could help bridge the gap. Telehealth appears to be an essential tool for patients with chronic conditions, as indicated by its association with the Charlson Comorbidity Index. Expanding remote monitoring capabilities could enhance care delivery for these populations.

As our findings showed, all observed differences remained significant after controlling for all sociodemographic determinants. These findings underscore the importance of an intersectional lens in understanding telehealth usage. Patterns of use are shaped not only by individual characteristics, such as age, gender, or health status, but by their intersections with cultural, geographic, and socioeconomic contexts.

For example, the markedly lower usage among older Arab, Bedouin, or Druze individuals that appears even after controlling for their tendency to reside in peripheral areas and their relatively younger age reflects how multiple, overlapping forms of marginalization (ethnicity, age, and geography) compound barriers to access. This finding supports research showing that digital health equity requires attention to multiple, overlapping social factors [29,93]. Effective strategies must address combined barriers rather than single issues.

Addressing disparities in telehealth adoption thus demands tailored, multidimensional strategies that go beyond single-axis solutions, ensuring culturally sensitive outreach, digital infrastructure development, and inclusive design of services.

### Health Equity Implications

The observed disparities in telehealth usage have significant implications for health equity. If digital health innovations disproportionately benefit those who are already advantaged, the young, socioeconomically privileged, and culturally dominant groups, they risk exacerbating existing health disparities rather than mitigating them.

Several mechanisms may link these usage disparities to health outcome inequities:

1. Delayed care: lower telehealth usage may lead to delayed care-seeking among vulnerable populations, potentially resulting in more advanced disease states at diagnosis.
2. Reduced preventive care: barriers to telehealth may reduce access to preventive services and early interventions.
3. Chronic disease management challenges: limited telehealth engagement may complicate ongoing management of chronic conditions, which are prevalent in this population (diabetes 17.7% and chronic pulmonary disease 15.2%)
4. Care fragmentation: differential adoption of telehealth versus in-person services may lead to fragmented care experiences for vulnerable populations.
5. Health care system strain: Inequitable telehealth distribution may increase in-person service demand among certain groups, straining physical health care resources.

These concerns are substantiated by Nouri et al [24], who demonstrated telehealth disparities associated with delayed care-seeking. Particularly concerning impacts on chronic disease management were documented [94,95], with Ojinnaka et al [96] showing care fragmentation increasing preventable hospitalizations among vulnerable populations.

### Strategic Interventions and Future Directions

The Israeli experience offers insights into addressing telehealth disparities. Obeid et al [97] found significant differences across population groups impacting adoption. Levin-Zamir et al [75] demonstrated that addressing language barriers requires considering intersections with age, digital literacy, and cultural preferences.

Understanding the complex relationship between sociodemographic characteristics, telehealth usage patterns, and health outcomes represents a critical research priority [98,99]. Such knowledge is essential for developing targeted interventions and policy approaches that can harness telehealth’s potential while ensuring its benefits are equitably distributed [24,35].

As telehealth continues to evolve as a fundamental component of health care delivery systems, addressing disparities becomes increasingly urgent for achieving health equity goals [83,100]. The lessons from Israel’s experience with health care implementation across diverse populations offer valuable guidance for developing more equitable digital health systems worldwide.

Evidence supports targeted interventions: Schifeling et al [101] documented digital literacy programs increasing adoption among older adults, while Chang et al [102] found multimodal approaches critical for safety net populations.

Strategic approaches should include culturally appropriate outreach, technology access programs, inclusive design principles, multimodal delivery methods, health care workforce training, continuous equity monitoring, and policy interventions prioritizing equitable access across all population segments.

### Community-Specific Telehealth Strategies

Based on our intersectional findings, tailored interventions must address the unique constellation of barriers facing different communities:

#### Arab or Bedouin Communities (82% Lower Odds of Use)

Our findings suggest that comprehensive cultural adaptation is essential. Interventions should include Arabic-language telehealth platforms with culturally appropriate interfaces, community health worker programs leveraging trusted local leaders, and gender-specific telehealth services addressing cultural preferences for same-gender providers. Partnership with existing community organizations and religious institutions for outreach, combined with family-centered telehealth models that align with cultural health care decision-making patterns, may address the multiple barriers identified.

#### Ultra-Orthodox Jewish Communities (Highest Adoption Rates)

Despite high usage, this community demonstrated unique patterns requiring specialized approaches. Expanding “kosher” telehealth devices that meet religious requirements, developing after-hours services accommodating religious observance schedules, and creating rabbinic endorsement programs for telehealth technologies could further optimize access. Privacy-enhanced platforms addressing community concerns about digital exposure should be prioritized.

#### Age-Intersected Interventions

The triple-rate difference between the youngest and oldest adults requires age-specific approaches that consider cultural context. For older Arab and Bedouin adults, combining cultural mediators with simplified technology training may address both digital literacy and cultural barriers. For younger adults in peripheral areas, leveraging mobile-first platforms and peer education models could optimize adoption. Family-supported telehealth models may be particularly effective for older adults across all communities.

#### Geographic-Socioeconomic Intersections

The U-shaped peripherality relationship suggests different intervention needs across geographic contexts. Medium peripheral areas showing the lowest adoption require targeted infrastructure investment combined with community-based digital literacy programs. Low-SES urban areas may benefit from device lending programs with multilingual technical support, while high-SES peripheral areas could use advanced telehealth services leveraging existing technological comfort.

### Limitations

Our study has several limitations. Despite using a comprehensive database, we could not control for provider-specific promotion or assess health and digital literacy factors that might better explain usage differences [75].

Our findings may have limited generalizability to the broader Israeli population, particularly regarding ultra-Orthodox communities, which comprised only 1.5% of our study population compared to 12%‐13% nationally. This underrepresentation may affect the applicability of our equity findings to these communities, despite our documentation of extensive telehealth use among the Religious Jewish population in our sample.

The focus on the Sharon-Shomron District may also limit broader applicability to other regions with different demographic compositions or health care infrastructure characteristics.

### Conclusions

This study demonstrates that the COVID-19 pandemic catalyzed a significant and lasting shift in telehealth usage patterns among CHS members. However, the benefits of this telehealth expansion have not been equally distributed across all population segments. Significant disparities exist along socioeconomic, cultural, age-related, and geographical lines.

The data present compelling evidence of significant disparities in telehealth usage across multiple sociodemographic dimensions. These disparities highlight the critical importance of applying equity lenses to digital health transformation efforts. Nonetheless, the extensive use of telehealth documented among the minority Ultra-Orthodox Jewish population demonstrates its potential to bridge gaps in health care access and provide tailored solutions, even for groups previously considered at risk of being negatively affected by technological developments.

The transformation of telehealth from a primarily after-hours alternative to an integrated component of regular health care delivery represents a fundamental shift in health care access patterns. As telehealth continues to evolve as a permanent feature of health care delivery systems, addressing the identified disparities will be crucial to ensuring that digital health advances promote rather than exacerbate health care equity.

Understanding complex relationships between sociodemographic characteristics, usage patterns, and health outcomes represents a critical research priority [98,99]. Such knowledge is essential for developing targeted interventions ensuring equitable benefit distribution [24,35].

While telehealth offers tremendous potential to improve health care access and outcomes, its benefits will only be fully realized if intentional efforts are made to ensure equitable adoption across all population segments. As health care systems continue to expand their digital footprints, they must simultaneously develop strategies to mitigate existing disparities and prevent the emergence of new inequities, also paying significant attention to intersectionality considerations. Only through such deliberate attention to equity concerns can digital health innovations fulfill their promise of more accessible, efficient, and effective health care for all members of society. The Israeli experience offers valuable guidance for developing more equitable digital health systems worldwide.

Future research should focus on understanding the specific barriers faced by low-adoption groups and evaluating the effectiveness of targeted interventions to increase equitable telehealth access across all population segments.

## Supplementary material

10.2196/77600 Multimedia Appendix 1 Variable definitions.

## Abbreviations

CHS: Clalit Health Services
HIPAA: Health Insurance Portability and Accountability Act
OR: odds ratio
SES: socioeconomic status
