# Supplementary material for: Telehealth Usage Disparities in Israel in Light of the COVID-19 Pandemic: Retrospective Cohort Study of Intersectional Sociodemographic Patterns and Health Equity Implications
Source: J Med Internet Res. 2025 Nov 27;27:e77600. doi: 10.2196/77600 (PMC12661909; doi:10.2196/77600)
Supplement: Multimedia Appendix 1 [file jmir-v27-e77600-s001.docx]

**Supplementary Materials: Variable Definitions**

**Socioeconomic Status (SES) Classification**

Socioeconomic status was classified using the Israel Central Bureau of Statistics (ICBS) neighborhood-level socioeconomic indices, which provide standardized measures of community-level socioeconomic characteristics. The ICBS methodology incorporates multiple indicators to create composite socioeconomic rankings:

**Classification Criteria:**

**Income Indicators:**

- Average household income
- Percentage of households receiving income support
- Percentage of households below poverty line

**Education Indicators:**

- Percentage of adults with academic education (bachelor's degree or higher)
- Percentage of adults with secondary education completion
- Average years of schooling

**Employment Indicators:**

- Labor force participation rates
- Unemployment rates
- Percentage employed in professional/managerial occupations

**Housing Quality Indicators:**

- Average number of persons per room
- Percentage of households with standard housing amenities
- Housing ownership rates

**SES Categories:**

- **Low SES:** Bottom tertile of ICBS socioeconomic index (clusters 1-3 on 1-10 scale)
- **Medium SES:** Middle tertile of ICBS socioeconomic index (clusters 4-6 on 1-10 scale)
- **High SES:** Top tertile of ICBS socioeconomic index (clusters 7-10 on 1-10 scale)

**Residency Type Classifications**

**Kibbutz**

Collective agricultural communities based on principles of joint ownership, shared labor, and communal living. Members traditionally share resources, decision-making, and economic outcomes. Modern kibbutzim may have varying degrees of privatization while maintaining cooperative principles.

**Characteristics:**

- Collective ownership of property and means of production
- Democratic governance through general assembly
- Historically agricultural focus, now often diversified
- Typically 50-2,000 residents
- Emphasis on equality and mutual aid

**Moshav/Kfar**

Small agricultural communities with individually owned farms but cooperative purchasing and marketing arrangements. Represents a middle ground between collective kibbutz and private farming.

**Moshav Characteristics:**

- Individual family farms with private ownership
- Cooperative purchasing of supplies and marketing of products
- Mutual aid and shared services (machinery, irrigation)
- Democratic governance with elected committees
- Typically 100-1,000 residents

**Kfar Characteristics:**

- Rural villages with mixed agricultural and residential character
- Individual property ownership
- Local council governance
- May include both farming and non-farming residents

**Moatza/Ayara**

Regional councils (Moatza) and local authorities (Ayara) representing smaller municipal entities outside major urban centers.

**Moatza (Regional Council) Characteristics:**

- Governing body for multiple small communities in a geographic region
- Provides municipal services to rural and semi-rural areas
- Covers settlements, moshavim, kibbutzim, and small towns
- Population typically ranges from 2,000-20,000 across the entire region

**Ayara (Local Authority) Characteristics:**

- Local government for smaller towns and communities
- Population typically 1,000-10,000 residents
- Provides basic municipal services
- Often rural or semi-rural character

**Non-Jewish Settlement**

Communities primarily inhabited by Arab, Bedouin, Druze, or other non-Jewish populations, often with distinct cultural, linguistic, and social characteristics.

**Characteristics:**

- Distinct cultural and linguistic communities
- May have traditional governance structures alongside formal municipal government
- Often face unique infrastructure and service delivery challenges
- Variable socioeconomic conditions

**Periphery Classification Methodology**

Periphery classification is based on the Israel Central Bureau of Statistics methodology that considers geographic distance from major urban centers and availability of services and infrastructure.

**Classification Criteria:**

**Geographic Factors:**

- Distance to nearest major city (>100,000 population)
- Travel time to central metropolitan areas (Tel Aviv, Jerusalem, Haifa)
- Transportation infrastructure availability
- Road connectivity and public transportation access

**Service Availability:**

- Healthcare facility accessibility (hospitals, specialized clinics)
- Educational institution availability (universities, vocational training)
- Commercial and banking services
- Government service offices

**Infrastructure Development:**

- Telecommunications infrastructure
- Utilities reliability (electricity, water, sewage)
- Internet connectivity quality
- Industrial and employment centers proximity

**Periphery Categories:**

**Very Central:**

- Located within or adjacent to major metropolitan areas
- Excellent transportation connectivity
- Full range of services and infrastructure readily available
- Examples: Tel Aviv suburbs, Jerusalem suburbs

**Central:**

- Close proximity to major urban centers
- Good transportation links
- Most services available locally or within reasonable distance
- Well-developed infrastructure

**Medium Peripheral:**

- Moderate distance from major urban centers
- Some transportation and service limitations
- Basic services available but specialized services may require travel
- Mixed infrastructure development

**Peripheral:**

- Significant distance from major urban centers
- Limited transportation options
- Reduced service availability
- Infrastructure gaps

**Very Peripheral:**

- Remote locations with substantial distance from urban centers
- Limited transportation and connectivity
- Significant service access challenges
- Infrastructure limitations
- Examples: Negev desert communities, northern border areas

**Data Sources:**

All classifications are based on official Israel Central Bureau of Statistics designations and are updated periodically to reflect demographic and infrastructure changes. These classifications are used for government planning, resource allocation, and policy development purposes.
